# Supplementary material for: Adaptive QP algorithm for depth range prediction and encoding output in virtual reality video encoding process
Source: PLoS One. 2024 Sep 25;19(9):e0310904. doi: 10.1371/journal.pone.0310904 (PMC11424003; doi:10.1371/journal.pone.0310904)
Supplement: S1 Data — (DOCX) [file pone.0310904.s001.docx]

**Table1 Data from Figure 8**

| Bitratekbp | | (20000,40000) | (40000,60000) | (60000,80000) | (80000,100000) |
| --- | --- | --- | --- | --- | --- |
| Test sequence 1 | JVET | (37.1,40.1) | (40.1,42.1) | (42.1,44.3) | (44.3,45.0) |
|  | Research algorithms | (37.2,40.2) | (40.2,42.2) | (42.1,44.4) | (44.4,44.9) |
| Test sequence 2 | JVET | (35.1,39.1) | (39.1,40.3) | (40.3,43.3) | (43.3,44.6) |
|  | Research algorithms | (35.2,39.2) | (39.2,40.4) | (40.4,43.4) | (43.4,44.7) |
| Test sequence 3 | JVET | (35.4,39.6) | (35.1,42.4) | (42.4,43.7) | (43.7,45.0) |
|  | Research algorithms | (35.5,39.7) | (35.2,42.5) | (42.5,43.8) | (43.8,45.0) |
| Test sequence 4 | JVET | (35.1,38.6) | (38.6,42.5) | (42.5,43.4) | (43.4,44.6) |
|  | Research algorithms | (35.2,38.7) | (38.7,42.6) | (42.6,43.5) | (43.5,44.7) |

**Table2 Data from Figure 9**

|  | | Depth 0 | Depth 1 | Depth 2 | Depth 3 |
| --- | --- | --- | --- | --- | --- |
| Part 1 | Urban charm | 82.0 | 83.6 | 89.4 | 94.1 |
|  | Electrode Polt | 87.5 | 82.8 | 87.6 | 93.2 |
|  | Castle | 82.7 | 85.6 | 91.7 | 85.2 |
| Part 2 | Landing | 93.6 | 86.8 | 92.1 | 95.0 |
|  | Square Lane | 83.2 | 84.9 | 87.2 | 94.3 |
|  | Balboa | 86.7 | 84.9 | 90.2 | 93.6 |
| Part 3 | Gas lamp | 87.5 | 84.3 | 92.4 | 95.8 |
|  | Wheelbarrow | 93.7 | 87.1 | 90.3 | 95.8 |
|  | Port | 86.9 | 86.4 | 92.4 | 96.5 |
|  | Kite flying | 85.2 | 87.6 | 92.4 | 93.8 |
|  | Tramcar | 83.7 | 86.4 | 92.4 | 95.8 |
|  | Scooter | 85.3 | 87.7 | 92.4 | 96.9 |
|  | Average | 87.1 | 86.6 | 92.1 | 95.8 |

**Table3 Data from Figure 10**

| Method | Division accuracy(%) |
| --- | --- |
| LCEVC-Example 1 | 82.5 |
| Research algorithms-Example 1 | 93.4 |
| LCEVC-Example 2 | 83.4 |

**Table4 Data from Figure 11**

| Image width | 400 | 800 | 1200 | 1600 |
| --- | --- | --- | --- | --- |
| Weight value | 0.76 | 1.37 | 1.08 | 0.08 |

**Table5 Data from Figure 12**

| Bitrate(kbps) | | (10000,150000) | (150000,200000) | (200000,250000) | (250000,300000) |
| --- | --- | --- | --- | --- | --- |
| Test sequence 1 | VVC | (42.6,43.5) | (43.5,45.0) | (43.5,46.2) | (46.2,47.1) |
|  | Research algorithms | (42.7,43.6) | (43.5,45.1) | (45.1,46.3) | (46.3,47.2) |
| Test sequence 2 | VVC | (44.5,46.7) | (46.7,47.5) | (47.5,48.0) | (48.0,48.2) |
|  | Research algorithms | (44.6,46.8) | (46.8,47.6) | (47.6,48.1) | (48.1,48.3) |
| Test sequence 3 | VVC | (43.6,44.2) | (44.2,45.1) | (45.1,46.4) | (46.4,46.6) |
|  | Research algorithms | (43.7,44.3) | (44.3,45.2) | (45.2,46.5) | (46.5,46.7) |
| Test sequence 4 | VVC | (43.6,44.5) | (44.5,45.2) | (45.2,45.5) | (45.5,47.6) |
|  | Research algorithms | (43.7,44.5) | (44.5,45.3) | (45.3,45.6) | (45.6,47.7) |
