# Supplementary material for: Adaptive QP algorithm for depth range prediction and encoding output in virtual reality video encoding process
Source: PLoS One. 2024 Sep 25;19(9):e0310904. doi: 10.1371/journal.pone.0310904 (PMC11424003; doi:10.1371/journal.pone.0310904)
Supplement: Attachment — Submitted filename: Response to comments.docx [file pone.0310904.s002.docx]

Reviewers' comments:

Reviewer's Responses to Questions

Comments to the Author

1. Is the manuscript technically sound, and do the data support the conclusions?

The manuscript must describe a technically sound piece of scientific research with data that supports the conclusions. Experiments must have been conducted rigorously, with appropriate controls, replication, and sample sizes. The conclusions must be drawn appropriately based on the data presented.

Reviewer #1: Yes

2. Has the statistical analysis been performed appropriately and rigorously?

Reviewer #1: Yes

3. Have the authors made all data underlying the findings in their manuscript fully available?

The PLOS Data policy requires authors to make all data underlying the findings described in their manuscript fully available without restriction, with rare exception (please refer to the Data Availability Statement in the manuscript PDF file). The data should be provided as part of the manuscript or its supporting information, or deposited to a public repository. For example, in addition to summary statistics, the data points behind means, medians and variance measures should be available. If there are restrictions on publicly sharing data—e.g. participant privacy or use of data from a third party—those must be specified.

Reviewer #1: Yes

4. Is the manuscript presented in an intelligible fashion and written in standard English?

PLOS ONE does not copyedit accepted manuscripts, so the language in submitted articles must be clear, correct, and unambiguous. Any typographical or grammatical errors should be corrected at revision, so please note any specific errors here.

Reviewer #1: Yes

5. Review Comments to the Author

Please use the space provided to explain your answers to the questions above. You may also include additional comments for the author, including concerns about dual publication, research ethics, or publication ethics. (Please upload your review as an attachment if it exceeds 20,000 characters)

Reviewer #1: This paper proposes an efficient way to encode 360-degree videos, using adaptive QP algorithm.

Polar-region aware QP adaptation algorithm is well known researches.

It seems that more novel method should be proposed for paper acceptance.

Reply: Thank you very much for yoursuggestions.The technical uniqueness and application of the current methods have been explained in the Related Works Section. The authors have placed the innovative aspects of our research in the Related Works Section. By comparing with the research of other scholars, it can be concluded that the innovation of this study can also have a reasonable logic.

Compared with other studies, this study has innovation in the application of technology and language. Firstly, the encoding unit segmentation depth prediction method based on proximity depth correlation is applied in the model. This method quickly predicts the ideal segmentation depth of encoding units by evaluating image attributes, greatly reducing complexity. Secondly, in terms of mode selection strategy, the model will adaptively select modes in different video regions based on video content, which effectively improves encoding efficiency. Finally, in the adaptive QP algorithm, the weighted spherical peak signal-to-noise ratio weighting method is introduced for adaptive adjustment. It can adaptively adjust the viewing priority of different regions of spherical videos to improve image quality. Given the technical characteristics of the model, it can be applied to high-quality virtual reality spherical videos under large-scale data transmission. This model can reduce the amount of data transmission while ensuring image quality, thereby supporting large-scale real-time playback of virtual reality videos.

Of course, the proposed method showed gain compared to the anchor.

Experiments on EVC, VVC, AV1, and VP9 are convincing.

More explanations on Fig. 8 should be added, especially which software is used for JVET (is it VVC or exploration test model?).

Reply: Thank you for yoursuggestions. VVC software is used for JVET in this study. The software used in the study and the in-depth analysis in Figure 8 have been supplemented. As follows:

This study used VVC software. As a new video encoding technology, VVC has more advantages in image quality compared to traditional technologies such as H.264/AVC and H.265/HEVC. It can compress at least 40% of the data while maintaining the same image quality. However, VVC has a limitation that it requires high encoding and decoding resources when dealing with complex video sequences or scenes. This leads to a decrease in the application efficiency of the model under resource constraints. However, from Figure 8, it can be seen that the research designed method can provide better encoding efficiency while maintaining the same image quality. As the quality continues to improve, it shows a late curve in Figure 8. At this time, as the range of quality fluctuations narrows, the bit rate growth rate of each sequence gradually slows down, indicating that the model can achieve high-quality results with smaller bit rate increments. It can be seen that it has efficiency advantages in processing high-quality videos.

6. PLOS authors have the option to publish the peer review history of their article (what does this mean?). If published, this will include your full peer review and any attached files.

If you choose “no”, your identity will remain anonymous but your review may still be made public.

Do you want your identity to be public for this peer review? For information about this choice, including consent withdrawal, please see our Privacy Policy.

Reviewer #1: No
