# Supplementary material for: Adaptive QP algorithm for depth range prediction and encoding output in virtual reality video encoding process
Source: PLoS One. 2024 Sep 25;19(9):e0310904. doi: 10.1371/journal.pone.0310904 (PMC11424003; doi:10.1371/journal.pone.0310904)
Supplement: Attachment — Submitted filename: Response to comments.docx [file pone.0310904.s003.docx]

Reviewers' comments:

Reviewer's Responses to Questions

Comments to the Author

1. If the authors have adequately addressed your comments raised in a previous round of review and you feel that this manuscript is now acceptable for publication, you may indicate that here to bypass the “Comments to the Author” section, enter your conflict of interest statement in the “Confidential to Editor” section, and submit your "Accept" recommendation.

Reviewer #1: All comments have been addressed

Reply: Thank you very much.

2. Is the manuscript technically sound, and do the data support the conclusions?

The manuscript must describe a technically sound piece of scientific research with data that supports the conclusions. Experiments must have been conducted rigorously, with appropriate controls, replication, and sample sizes. The conclusions must be drawn appropriately based on the data presented.

Reviewer #1: Partly

Reply: To reduce coding complexity, a fast partitioning strategy for coding units was designed, and the performance of the model was tested using coding efficiency indicators. To improve coding performance, an adaptive algorithm was designed, which takes into account the different levels of user attention in 360 degree virtual reality videos. Different adaptive quantization parameter adjustment strategies were adopted for the equatorial and polar regions, and model performance was tested using two indicators: bit rate reduction rate and weighted peak signal-to-noise ratio. In order to make the experiment more rigorous, a horizontal comparison experiment was added, and the dataset was compared with the horizontal model. Improve performance analysis while improving the reducibility of experiments. Please see details in Tables 1, 2, 5 and their explanations.

After revision, the experiments was conducted rigorously, with appropriate controls, replication, and sample sizes. The conclusions were drawn appropriately based on the data presented.

3. Has the statistical analysis been performed appropriately and rigorously?

Reviewer #1: Yes

Reply: Thank you very much.

4. Have the authors made all data underlying the findings in their manuscript fully available?

The PLOS Data policy requires authors to make all data underlying the findings described in their manuscript fully available without restriction, with rare exception (please refer to the Data Availability Statement in the manuscript PDF file). The data should be provided as part of the manuscript or its supporting information, or deposited to a public repository. For example, in addition to summary statistics, the data points behind means, medians and variance measures should be available. If there are restrictions on publicly sharing data—e.g. participant privacy or use of data from a third party—those must be specified.

Reviewer #1: (No Response)

Reply: Thank you very much for your comments. All data underlying the findings have been provided.

5. Is the manuscript presented in an intelligible fashion and written in standard English?

PLOS ONE does not copyedit accepted manuscripts, so the language in submitted articles must be clear, correct, and unambiguous. Any typographical or grammatical errors should be corrected at revision, so please note any specific errors here.

Reviewer #1: (No Response)

Reply: Thank you very much for your comments. The language in the manuscript was clear, correct, and unambiguous. And there was no typographical or grammatical error after revision.

6. Review Comments to the Author

Please use the space provided to explain your answers to the questions above. You may also include additional comments for the author, including concerns about dual publication, research ethics, or publication ethics. (Please upload your review as an attachment if it exceeds 20,000 characters)

Reviewer #1: 1. In Fig. 8, typo: WA-PSNR -> WS-PSNR.

Reply: Thank you for your comments. The WA-PSNR in Figure 8 has been changed to WS-PSNR.

**Fig.8 Comparison of deep partitioning models**

2. In Related Works, what does it mean by "Language"? In the following sentence:

...Compared with other studies, this study has innovation in the application of technology and language...

Reply: Thank you very much for your comments. The sentence was changed to “Compared with other studies, this study has innovation in the application of technology.”

3. Depth range prediction (DRP) method is not well explained in the paper.

Reply: Thank you very much for your comments. More explanation has been provided in section 2.1, as follows:

Depth range prediction is a technique used to optimize the virtual reality video encoding process, with the main purpose of improving encoding efficiency and reducing computational complexity by predicting the optimal segmentation depth of the coding unit (CU) in advance. This study achieved self-adaptive depth range prediction through four steps, including adjacent block depth analysis, cost function definition, depth prediction strategy design, and simplified segmentation process. Analyze the depth information of adjacent blocks through depth range prediction, predict the segmentation depth of the target CU in advance, simplify the encoding process, and reduce computational complexity.

4. In the following sentence, "serial number" is not well explained.

...In formula (12), index represents the serial number, N...

Reply: Thank you for your comments. In formula (12), $index\cdot N$ represents the serial number.

5. In formula (10), why 3log2(w) was used? It would be better with explanation of this.

Reply: Thank you for your suggestion. The original formula (10) has been changed to formula (9). The 3log2 (w) in formula (9) has been explained as follows:

In formula (10), $3\cdot LOG_{2}\left(w\right)$ represents binary logarithm, and coefficient 3 is an empirical parameter.

6. In Fig. 8, enlarged section of RD-curve needs to be added for the following sentence:

...As the quality level improves, the slow rise of the BRRR curve in the later stage may mean that as the quality further improves, the required increase in bit rate is less...

Reply: Thank you for your suggestion. In Figure 8, we have added a comparison of the amplification slope direction and corresponding analysis, as follows:

**Fig.8 Comparison of deep partitioning models**

As the quality level improves, the slow rise of the BRRR curve in the later stage may mean that as the quality further improves, the required increase in bit rate is less. From the direction of the enlarged slope line, it can be seen that the slope of the later line is significantly smaller, indicating a significant decrease in the growth rate of the indicator.

7. In Fig. 9, Part 1, 2, 3 are ambiguous.

Reply: Thank you for your suggestion. The subheadings in Figure 9 have been changed as follows:

**Fig.9 Division accuracy**

8. In Fig. 9, regarding the depth, trade-off should exist between accuracy and encoding time for different depth values.

In later experiments, which depth value was used?

Reply: Thank you for your suggestion. Depth 3 has been selected, and the specific explanation is as follows:

This study defines a novel cost function, namely the CU depth error, as a new evaluation criterion for the complexity of CU textures. Its calculation depends on the segmentation depth of surrounding blocks and the texture characteristics of the target block.Depth represents the reference depth and is usually divided into four numerical cases, namely 0, 1, 2, and 3. represents the number of blocks, and represents the depth value corresponding to the blocks. The depth division is Figure 4. Among all data topics, the accuracy of Depth 0 is generally distributed between 80% and 95%, the accuracy of Depth 1 is generally distributed between 80% and 90%, the accuracy of Depth 2 is generally distributed around 90%, and the accuracy of Depth 3 is generally distributed around 95%. Overall, the accuracy of Depth 0 and Depth 1 is relatively similar, with Depth 3 having the highest level of accuracy. From this, it can be seen that the higher the Depth value, the more capable the model is in capturing information details..

7. PLOS authors have the option to publish the peer review history of their article (what does this mean?). If published, this will include your full peer review and any attached files.

Reply: Thank you for your suggestion. The complete peer review has been added. Thank you again.
